# Antarctica: The final frontier for marine biological invasions

**DOI:** 10.1111/gcb.14600

**Published:** 2019-04-23

**Authors:** Arlie H. McCarthy, Lloyd S. Peck, Kevin A. Hughes, David C. Aldridge

**Affiliations:** ^1^ Department of Zoology University of Cambridge Cambridge UK; ^2^ British Antarctic Survey, NERC Cambridge UK; ^3^ BioRISC, St Catharine's College Cambridge UK

**Keywords:** alien species, biofouling, climate change, introduced species, invasion pathways, marine ecosystems, shipping, Southern Ocean

## Abstract

Antarctica is experiencing significant ecological and environmental change, which may facilitate the establishment of non‐native marine species. Non‐native marine species will interact with other anthropogenic stressors affecting Antarctic ecosystems, such as climate change (warming, ocean acidification) and pollution, with irreversible ramifications for biodiversity and ecosystem services. We review current knowledge of non‐native marine species in the Antarctic region, the physical and physiological factors that resist establishment of non‐native marine species, changes to resistance under climate change, the role of legislation in limiting marine introductions, and the effect of increasing human activity on vectors and pathways of introduction. Evidence of non‐native marine species is limited: just four marine non‐native and one cryptogenic species that were likely introduced anthropogenically have been reported freely living in Antarctic or sub‐Antarctic waters, but no established populations have been reported; an additional six species have been observed in pathways to Antarctica that are potentially at risk of becoming invasive. We present estimates of the intensity of ship activity across fishing, tourism and research sectors: there may be approximately 180 vessels and 500+ voyages in Antarctic waters annually. However, these estimates are necessarily speculative because relevant data are scarce. To facilitate well‐informed policy and management, we make recommendations for future research into the likelihood of marine biological invasions in the Antarctic region.

## Graphical abstract

Are there non‐native marine species in Antarctica? With over 500 visits from more than 180 vessels annually and rapidly changing environmental conditions, Antarctica appears to be increasingly vulnerable to impacts from non‐native marine species. We explore factors that influence the likelihood of non‐native marine species establishing in the Antarctic region, present new estimates for human activity, and make recommendations to researchers, environmental managers and policy makers.

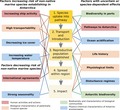

## 1 INTRODUCTION

Invasive non‐native species drive ecological changes that impact biodiversity and ecosystem services in almost all environments (Bax, Williamson, Aguero, Gonzalez, & Geeves, 2003; Chan & Briski, 2017; Lowe, Browne, Boudjelas, & De Poorter, 2004; Pimentel, Zuniga, & Morrison, 2005). The need to mitigate impacts of invasive species and understand their pathways of introduction is a global priority (CBD, n.d.; Roy et al., 2014; Sutherland et al., 2014, 2017, 2018). Invasive marine species can alter habitat structure, community composition, food webs and community dynamics (Como et al., 2016; Goren, Galil, Diamant, Stern, & Levitt‐Barmats, 2016; Lutz‐Collins, Cox, & Quijón, 2016; Oug, Cochrane, Sundet, Norling, & Nilsson, 2011; Oug, Sundet, & Cochrane, 2018; Palomo, Bagur, Quiroga, Soria, & Bugnot, 2016), which can lead to, for example, fisheries collapse and diminished ecosystem services (Grosholz et al., 2000; Johnston, Purkis, & Dodge, 2015). Although marine species, particularly invasive species, can disperse across large spatial scales (Kinlan & Gaines, 2003), polar regions possess few marine invaders (Molnar, Gamboa, Revenga, & Spalding, 2008; Ruiz & Hewitt, 2009). The Arctic has 34 recorded non‐native marine species (NNMS) from 54 introduction events (Chan et al., 2019). In contrast, Antarctica has no confirmed populations of NNMS and reports of only five free‐living marine species that were potentially transported by anthropogenic means: *Ulva intestinalis *(cryptogenic, grass kelp), *Hyas araneus *(great spider crab), *Bugula neritina *(brown bryozoan), *Ciona intestinalis *(vase tunicate), *Ectopleura crocea *(pinkmouth hydroid). Nonetheless, the need to recognize and mitigate human influences, including non‐native species, is a pressing issue for Antarctic research (Kennicutt et al., 2014, 2015; Rintoul et al., 2018). Given the high levels of endemism and unique taxonomic combinations within Antarctic ecosystems, changes in Antarctic biodiversity are recognized as globally important conservation priorities (Sutherland et al., 2012, 2011, 2015). Although NNMS in the Antarctic region are rare and historically have been of little concern, climate change and increasing human activity are expected to increase the establishment and potential impact of NNMS.

Climate change in Antarctic environments and human activity in the region are recognized as major factors increasing the risk of invasion by terrestrial species and likely for marine species, too (Frenot et al., 2005; Galera, Chwedorzewska, Korczak‐abshire, & Wódkiewicz, 2018; Hughes & Convey, 2010; McGeoch, Shaw, Terauds, Lee, & Chown, 2015). Thus far, terrestrial non‐native species have faced substantial natural barriers (Chown et al., 2012) that are now weakening through changing climate and increasing human activity (Duffy et al., 2017). In the marine realm, barriers to invasion can be physical, such as water currents and ice cover, or physiological, for example species’ lower thermal limits and specific life histories. However, future climate scenarios predict increased availability of suitable habitat for some coastal species, both native and non‐native (Byrne, Gall, Wolfe, & Agüera, 2016; Griffiths, Meijers, & Bracegirdle, 2017). In addition, human activity from ships may have increased 5‐ to 10‐fold since the 1960s around parts of Antarctica, especially along the Antarctic Peninsula (see Section 2.4; Aronson, Thatje, McClintock, & Hughes, 2011; Bender, Crosbie, & Lynch, 2016; Steig et al., 2009). The combination of climate change and increasing human activity will likely lower the physical and physiological barriers to invasion by NNMS.

**Figure 1:**
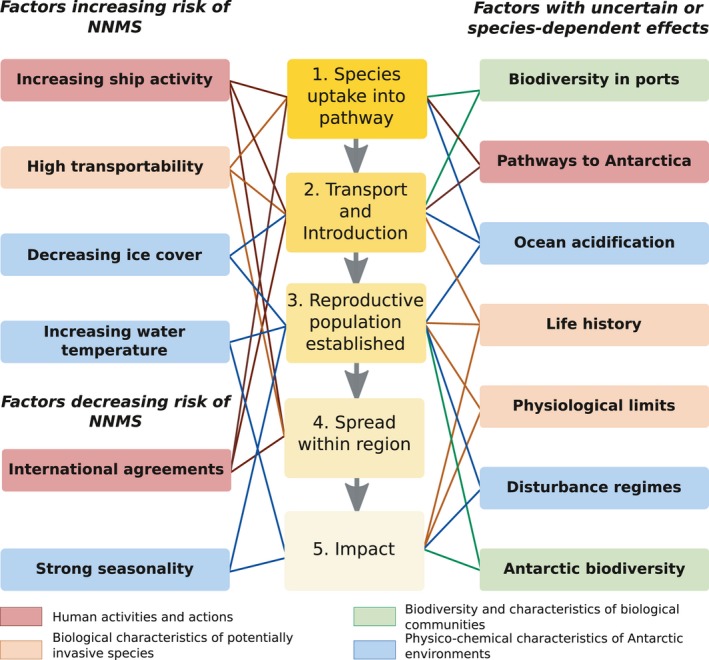
Factors influencing the risk of non‐native marine species (NNMS) becoming invasive in Antarctica and the Southern Ocean throughout the 5 main stages of invasion process. Factors can be anthropogenic or related to a species’ physiology, biodiversity or the abiotic environment. Factors and how they relate to stages of the invasion process are discussed in more detail in the main text

The likelihood of a non‐native species being transported to a new environment is influenced by a complex inter‐related range of natural and anthropogenic factors that affect different stages of the invasion process (Figure 1). In this review we investigate the key factors presented in Figure 1 that affect the potential for NNMS to become invasive in the Antarctic region now and in the future. In doing so, we:

1. present current knowledge, especially on factors affecting stages 1 and 2 of the invasion process, which includes a list of observations of NNMS from the Antarctic region and new estimates of ship activity around Antarctica and the Southern Ocean;
2. discuss factors currently affecting stage 3 of the invasion process;
3. discuss how climate change is altering the factors that predominantly affect stage 3 of the invasion process;
4. detail the international agreements and governance structure relevant to NNMS in the Antarctic region, which affect multiple stages of the invasion process;
5. discuss the future of human activity in the Antarctic region in relation to anthropogenic introductions;
6. make recommendations for Antarctic researchers, environmental managers and policy makers.

## 2 CURRENT KNOWLEDGE OF NON‐NATIVE MARINE SPECIES AND THEIR TRANSPORT TO ANTARCTICA

For the Antarctic region, uptake of NNMS into pathways and their subsequent transport and introduction (stages 1 and 2 of the invasion process; Figure 1) have been the focus of most research. Despite this, the presence of NNMS in the Antarctic region, their vectors of transport, and their pathways are still poorly understood. Here, we discuss current information and present new data on key factors affecting stages 1 and 2 of the invasion process (Figure 1): transportability of potential NNMS, pathways to Antarctica via different vectors, biodiversity in ports and increasing ship activity.

### 2.1 Transportability – Evidence of NNMS being transported to Antarctica

Knowledge of the transport of NNMS to Antarctica is extremely limited. To understand highly “transportable” species' uptake into pathways and transport to Antarctica (stages 1 and 2; Figure 1), we examined peer‐reviewed literature, publicly accessible grey literature and the Global Invasive Species Database (GISD) (Invasive Species Specialist Group ISSG, 2015) for records of NNMS around Antarctica and the sub‐Antarctic islands. We also identified species potentially poised to establish in Antarctica based on (a) their presence in fouling communities on Antarctic‐going vessels (Hughes & Ashton, 2017; Lee & Chown, 2007, 2009; Lewis, Hewitt, Riddle, & McMinn, 2003; Lewis, Riddle, & Hewitt, 2004; Lewis, Riddle, & Smith, 2005), (b) their current distributions in Ocean Biogeographic Information System (OBIS, 2018) and Global Biodiversity Information Facility (GBIF: The Global Biodiversity Information Facility, 2018), and (c) their known invasive capacity (presence in GISD or relevant literature). The resulting lists (Table 1, Table S1) reflect current knowledge of NNMS in the Antarctic region and within transport pathways but may not be exhaustive.

**Table 1:** Documented observations of non‐native marine species recorded around Antarctica and sub‐Antarctic islands, including possible anthropogenic introductions and observations from outside a species’ typical range. * indicates species that have been found fouling vessels that travel to Antarctica (surveys conducted outside the Southern Ocean). Underlined species are invasive in part of their range according to the Global Invasive Species Database (Invasive Species Specialist Group ISSG, 2015). Global range is based on records in the Ocean Biogeographic Information System (OBIS, 2018) and the Global Biodiversity Information Facility (GBIF: The Global Biodiversity Information Facility, 2018). SSI = South Shetland Islands, located at the northern tip of the Antarctic Peninsula. Species are listed using the most recent accepted name in the World Register of Marine Species (Horton et al., 2018), which may differ from the name in the original publication.

Taxa		Number of specimens (source)	Location of observation(s)	Year	Global Range	Likely dispersal mechanism
PLANTAE						
*	*Ulva intestinalis* Linnaeus, 1753 (gutweed, grass kelp)	Established in multiple locations (Clayton, Wiencke, & Klöser, 1997; Greenslade & Van Klinken, 1994; Kenny & Haysom, 1962; Pellizzari et al., 2017; Valentin, Dalto, & Gestinari, 2010)	Throughout SSI (Deception, Half Moon, Livingston, Robert, Nelson, King George, Penguin and Elephant Islands) and Macquarie Island	1962–2013	Cosmopolitan except high latitudes. Native in sub‐Antarctic including: Auckland Islands, Îles Kerguelen, Tierra del Fuego, Macquarie Island. Cryptogenic in South Shetland Islands	Natural (rafting), possibly hull fouling/ ballast^a^
anamalia						
**arthropoda**						
**Decapoda**						
Anomura						
	*Emerita* sp. Scopoli, 1777 (larval)	1 (Thatje & Fuentes, 2003)	King George Island (SSI) (62°14′33″S, 58°43′8″W)	2002	Southern limit of range in northern Chile and southern Brazil	Natural (passive planktonic)^b^
Brachyura						
	*Hyas araneus *Linnaeus, 1758 (great spider crab)	2 (Tavares & De Melo, 2004)	Elephant Island (SSI) (61°05′93″S, 55°47′07″W)	1986	North Atlantic	Ballast^c^
	*Rochinia gracilipes *A. Milne‐Edwards, 1875	1 (Griffiths et al., 2013)	SSI		Brazilian coast	Unknown^d^
	*Halicarcinus planatus *(Fabricius, 1775)	2 (Aronson et al., 2015; Griffiths et al., 2013; Stebbing, 1915)	Deception Island (SSI, 62°57′S, 60°29′W) and South Orkney Islands (60°41′S, 45°12′W)	2010, c. 1914	Southern South America and sub‐Antarctic Islands	Unknown^e^
	*Pinnotheres* sp. Bosc, 1801 (larval)	1 (Thatje & Fuentes, 2003)	King George Island (SSI), (62°14′33″S, 58°43′81″W)	2002	South America. Southern limit of natural range in Northern Argentina and Chiloe Island	Natural (passive planktonic)^b^
**bryozoa**						
*	*Bugula neritina* Linnaeus, 1758 (brown bryozoan, common bugula)	1 (Griffiths, Linse, & Crame, 2003)	Off Dronning Maud Land (70°19′54″S, 24°13′30″E), East Antarctica	1960	Cosmopolitan except high latitudes (native range unknown)	Hull fouling^f^
**chordata**						
*	*Ciona intestinalis*Linnaeus, 1767 (vase tunicate)	1 (Gutt, Sirenko, Arntz, Smirnov, & De Broyer, 2000)	Off Dronning Maud Land (71°8′18″S, 11°32′24″W), East Antarctica	1996	Native to North Atlantic, Mediterranean, widespread as non‐native species, including the Arctic	Hull fouling^f^
**cnidaria**						
*	*Ectopleura crocea* Agassiz 1862 (pinkmouth hydroid)	43 (Griffiths et al., 2003)	Off Dronning Maud Land (approx. 71°7′S, 11°28′W) and off Queen Mary Land (approx. 66°31′S, 95°58′E), East Antarctica	1996	Native range NE Atlantic, widespread as non‐native species	Hull fouling^f^

a The status of *U. intestinalis *in the Antarctic region is uncertain. Clayton et al. suggested *U. intestinalis* may have been anthropogenically introduced, but subsequent publications have not mentioned potential non‐native status of the species in the South Shetland Islands, and it appears to be considered native to sub‐Antarctic islands.

b Considered non‐native (i.e. outside natural range) by source reference but unlikely to have been transported anthropogenically.

c Based on discussion in source references.

d No discussion of dispersal mechanism in source reference.

e Both natural and anthropogenic dispersal mechanisms raised in source references.

f Based on status as a common hull fouling organism that has been transported elsewhere via hull fouling.

We found six studies that have examined the hulls of ships active in the Southern Ocean: six research vessels, two fishing vessels and one yacht (Hughes & Ashton, 2017; Lee & Chown, 2007, 2009; Lewis et al., 2003, 2004, 2005). Only one study has examined ballast water and found that it was all of Antarctic origin and contained live Baccilariophyta, Dinophyceae, Copepoda and ciliates upon release into waters near Hobart (Lewis et al., 2003).

To date, five non‐native marine species have been observed free‐living in Antarctic waters (south of 60°S) that were potentially introduced by anthropogenic means (Table 1), three of which (*C. intestinalis*, *B. neritina *and *E. crocea*) are known as invasive species elsewhere in their ranges and have been found on ships that travel to Antarctica (Table S1). Of the 55 taxa reported from the hulls of Antarctic‐going vessels (Table S1), 15 have distributions in or records from the Arctic or sub‐Antarctic, which may indicate a tolerance of environmental conditions similar to those found in Antarctica. Of the same 55 taxa, ten species are considered invasive in part of their range. Six species have both high latitude distributions and are considered invasive: bryozoans *B. neritina *and *Schizoporella unicornis*, ascidians *Ascidiella aspersa* and *C. intestinalis*, the hydroid *E. crocea*, and the mussel *Mytilus galloprovincialis*. These six species, along with others not yet observed within pathways to Antarctica, may have the potential to colonize Antarctic coasts and warrant further investigation.

### 2.2 Pathways to Antarctica – Ship movements and vectors for NNMS

Currently, biofouling on ships’ hulls is likely to be the most important vector for transporting species to Antarctica (Lewis et al., 2005, see also Section 2.4, 5), although in the future other vectors such as marine plastics may become a concern (see Section 2.4, 6). Even so, in polar regions, ships that pass through sea‐ice will have their hulls scraped, removing encrusting organisms on exposed areas, (Hughes & Ashton, 2017; Lee & Chown, 2009; Lewis et al., 2004) potentially influencing the transport and introduction stage of the invasion process (Figure 1). Protected “niche” areas on ships (such as sea chests, moon pools, outlet ports and internal seawater systems) provide a sheltered habitat that can harbour NNMS (Coutts & Dodgshun, 2007; Frey, Simard, Robichaud, Martin, & Therriault, 2014) and species that breed within internal systems or sea chests, such as *M. galloprovincialis *(Lee & Chown, 2007; Piola & Grandison, 2017), may release pelagic larval stages into surrounding waters. While biofouling in niche areas may be expected to be particularly important in polar regions, hull surveys in the Arctic found niche areas provided no particular protection for biofouling organisms (Chan, MacIsaac, & Bailey, 2016) and the capacity for niche areas to harbour NNMS on voyages to Antarctica is unclear. Moreover, ice‐scour does not affect all vessels. Ships visiting sub‐Antarctic islands or the northern Antarctic Peninsula in summer may not encounter any sea‐ice. Furthermore, vessels without ice‐class hulls and many tourist and military vessels may choose to avoid sea‐ice. Under such scenarios, fouling on the open hull may present an important introduction risk.

**Figure 2:**
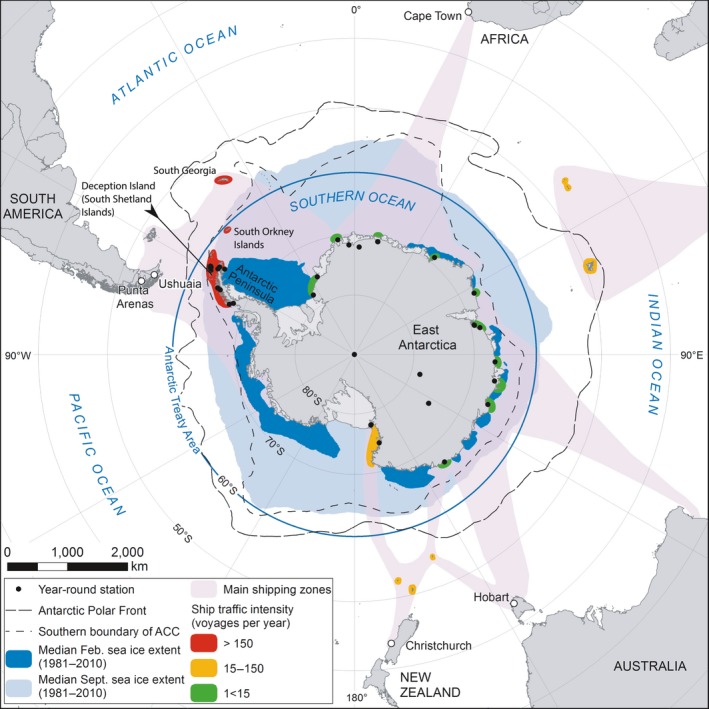
Map of Antarctica and Southern Ocean showing key features: Antarctic year‐round stations, sea‐ice extent in summer and winter, gateway ports, Antarctic Treaty area, Antarctic Circumpolar Current (ACC), areas of coastline within the Antarctic region with high, medium or low annual ship traffic. Estimates of ship traffic based on track density from MarineTraffic in 2016–2017 (MarineTraffic, 2018)

A ship's route to Antarctica and residence time in ports both outside and within the Southern Ocean will affect which non‐native species are transported, the number of propagules on a given ship and their likelihood of survival (stages 1 and 2 in Figure 1; Davidson, Brown, Sytsma, & Ruiz, 2009; Sylvester et al., 2011; Sylvester & MacIsaac, 2010). Despite the importance of these factors, potential vessel‐based pathways and routes into Antarctic waters for marine species are poorly understood, largely unquantified and urgently require research. Most journeys into Antarctic waters appear to pass through or begin at a so‐called “Gateway Port” (Figure 2), with examples including: Punta Arenas, Chile; Ushuaia, Argentina; Hobart, Australia; Christchurch, New Zealand; Cape Town, South Africa and other South Atlantic ports. Some Southern Ocean fishing operations depart from Port Louis, Mauritius (Austral Fisheries, 2018). Tourist vessels rarely stay in port for more than a few hours. In contrast, ships used by national operators for research or resupply, or both, may spend the winter at one port (Lewis et al., 2003), and can wait weeks between voyages and remain longer in Antarctic locations (Lee & Chown, 2007, 2009). Vessels operating at both poles, including both research and tourist vessels (AECO, 2018; IAATO, 2018b), may be at sea most of the year and bypass Southern Hemisphere gateway ports before their first Antarctic voyage of the season (G Adventures, 2019).

The most frequently travelled routes in the Southern Ocean connect South American and South Atlantic ports to the western Antarctic Peninsula, often via South Georgia and the South Orkney Islands (Figure 2) (Bender et al., 2016; Lynch, Crosbie, Fagan, & Naveen, 2010). This traffic services the research stations on the Antarctic Peninsula that account for 48% (37 of 77) of currently operational Antarctic stations (COMNAP, 2018a). The Antarctic Peninsula is also a hotspot for tourism and received 99% of Antarctic tourism (south of 60°S, i.e. excluding South Georgia) in 2016–2017 and 97% in 2017–2018 (IAATO, 2018a). Perhaps unsurprisingly, the Antarctic Peninsula and off‐shore islands at its northern tip have more recordings of marine (Table 1) and terrestrial (Hughes, Pertierra, Molina‐Montenegro, & Convey, 2015; McGeoch et al., 2015) non‐native species than anywhere else in continental Antarctica. While this may reflect a sampling bias, it is nonetheless an area worthy of particular focus for a monitoring programme, as advocated by the CEP Non‐Native Species Manual (CEP, 2017) and Climate Change Response Work Programme (CEP, 2015) (see Section 2.4, 7).

**Figure 3:**
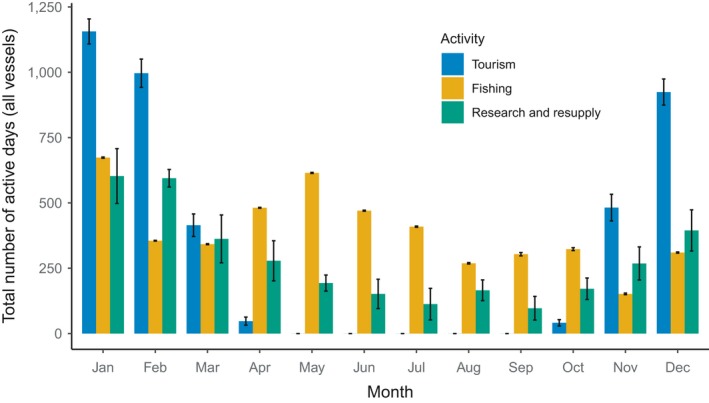
Monthly intensity of fishing, tourism and national Antarctic programmes (research and resupply) in the Antarctic region and the Southern Ocean. The total ship days per month for tourist vessels and research and resupply vessels are highly seasonal. For the fishing industry, the number of fishing days are presented, which is less than the total days spent at sea. Values are means ± *SE* for each month during the period 2012–2017. Mean annual ship days are 4,062 (Tourism), and 3,383 (Research and resupply). Mean annual fishing days is 4,703. Data on fishing activity was obtained from CCAMLR (2018a), and tourism activity from IAATO (2018a). Data on research and resupply was supplied by COMNAP (M. Rogan‐Finnemore, pers. comm., September 19, 2018)

Since marine propagule pressure in the Southern Ocean varies by season and vector (vessel) type, we used data provided by the International Association of Antarctica Tour Operators (IAATO, 2018a), the Commission for the Conservation of Antarctic Marine Living Resources (CCAMLR, 2017), and the Council of Managers of National Antarctic Programs (COMNAP, M. Rogan‐Finnemore, pers. comm., September 19, 2018) to examine the monthly intensity of ship activity across the tourism, fishing and research sectors for the period 2012–2017 (Figure 3). Data presented are based on the number of days at sea for tourism and research, but number of days fishing for fishing vessels. Therefore, the values for fishing vessels are likely conservative in comparison to research and tourism. Unless stated otherwise, all references to tourism refer to IAATO members and may not reflect all tourism activity in the region.

Tourist vessels operate from October to April with on average 4,062 ship days per year and repeatedly target the same coastal areas (Bender et al., 2016; Lynch et al., 2010). In 2017–2018, the most popular 10% (36 of 359) of tourist landing sites around Antarctica and sub‐Antarctic islands accounted for 67% of tourist visits and were locations in either the Antarctic Peninsula (30 of 36) or South Georgia (6 of 36) (IAATO, 2018a).

In contrast, fishing vessels operate throughout the year, with on average 4,703 active days per year (Figure 3). Fishing generally occurs offshore in the more northerly reaches of the CCAMLR areas during winter due to sea‐ice. Although fishing vessels venture south in summer, they generally stay further offshore than tourist vessels. As such, fishing vessels may pose a relatively low risk for introducing shallow benthic species. There is potential to move species between areas on fishing gear, and some fishing activities span sub‐Antarctic and Antarctic latitudes, but the risk of inter‐area transport of this sort in the Antarctic region is unknown.

Vessels used for research and resupply of stations in Antarctica are most active during the austral summer months (October to April) and represent on average 3,383 ship days per year (Figure 3). Research vessels tend to repeatedly visit the same coastal locations in Antarctica (the stations) and will remain alongside wharfs, in sheltered bays or alongside ice shelves or multiyear sea ice for periods of hours to days.

While the activity presented in Figure 3 represents most of the ship activity around Antarctica, there are other independent operators that are not part of national operations, IAATO or recognized fishing activity. These include ventures like the recent Antarctic Circumnavigation Expedition (ACE), which was the first project of the Swiss Polar Institute and supported a large number of international research projects at sites all around Antarctica between December 2016 and March 2017, and the 2019 Weddell Sea Expedition that used the icebreaker *SA Agulhas* to survey areas around the Larsen C ice shelf and to look for the wreck of Shackleton's *Endurance* in the Weddell Sea. They also include other activities including activist activities and cruises to support media activities, such as film crews for programmes such as the BBC's “Life in the Freezer”, which use a variety of vessels from private yachts to large icebreakers. Such private ventures south of 60°S must still be permitted by an appropriate national authority in accordance with Antarctic Treaty requirements and have completed an environmental impact assessment (see also Section 2.4, 5). While privately funded ventures are likely to be small in number compared to national operator activity and tourism, they may access sites rarely visited by the main operators, as was the case for the ACE cruise, and their importance for NNMS may require special consideration.

### 2.3 Biodiversity in gateway ports and regions visited by Antarctic‐going vessels

Ports serve as hubs or “bridgeheads” for vessel‐mediated transport of NNMS around the world (Ricciardi et al., 2017). Therefore, the native and non‐native biodiversity of gateway ports are key factors influencing the risk of transporting NNMS to Antarctica (Figure 1). Because of the high traffic and cool water temperature, native or non‐native species from southern South America could be likely candidates for colonization of coastal Antarctica. In particular, winter spawning species in gateway ports may have the opportunity to foul overwintering Antarctic‐going vessels, demonstrating how life history may influence stage 2 (transport and introduction, Figure 1). Yet in Argentina, there are relatively few NNMS in fouling communities in Ushuaia compared to other ports in Patagonia, perhaps due to environmental or biological factors (Schwindt et al., 2014). In Chile, no NNMS were found in a survey of ascidians in Punta Arenas and at sites up to 50 km away (Turon, Cañete, Sellanes, Rocha, & López‐Legentil, 2016), but non‐native *Mytilus* species hybridize with native mussel species in the Magellanic area of South America (Oyarzún, Toro, Cañete, & Gardner, 2016). *Mytilus *species have high latitude distributions in the northern hemisphere (Table S1, Telesca et al., 2018) and may warrant particular attention. However, very little is known about the lower thermal tolerances of native or non‐native South American species.

Seabed temperatures around continental and maritime Antarctica, though warming, will likely remain approximately 2–7°C colder than those in South American gateway ports for many decades (Griffiths et al., 2017), which may also point to high latitude Northern Hemisphere species as likely colonists and Arctic ports as possible sources of NNMS. Many Northern Hemisphere species have become invasive in southern temperate sites, for example in Port Phillip Bay (Melbourne), Australia (Hewitt et al., 2004), including some species with Arctic distributions. Although conditions in Port Phillip Bay are warmer than in either the Arctic or Southern Ocean, pathways to the Southern Hemisphere are clearly viable for some species of algae, polychaetes and sponges that can inhabit polar waters (Hewitt et al., 2004). Indeed, species with Arctic distributions have demonstrated capacity to adapt to polar conditions. While several species have reported natural bipolar distributions, being present at both poles but not in between, these are typically microorganisms (Montresor, Lovejoy, Orsini, Procaccini, & Roy, 2003; Pawlowski et al., 2007). Thus, observations of species with predominantly Arctic distributions, especially macrofauna or macroalgae, in the Antarctic region may well point to anthropogenic introductions. Although, the viability of anthropogenic trans‐polar pathways for NNMS is uncertain, the biodiversity of some Arctic locations could be a relevant factor for the transport of NNMS to the Antarctic region.

### 2.4 Increasing ship activity around Antarctica – Current estimates and historical comparisons

Estimates of human activity around Antarctica, especially from ships, are essential for quantifying propagule pressure along the pathways to Antarctica and identifying possibly source regions. As such, increasing ship activity is a key factor influencing stages 1 (species uptake), 2 (transport and introduction) and 4 (spread within Antarctic region) of the invasion process. Based on data published by IAATO (2018a, 2018b), CCAMLR (2017, CCAMLR (2018a), and COMNAP (2018b), we estimate that over 180 ships were active around Antarctica and the sub‐Antarctic islands in 2017–2018 on potentially 500+ voyages (return journeys of any duration) into the Southern Ocean or to sub‐Antarctic islands (Table 2). For comparison, 1960 saw approximately 30 vessels active on 75–100 voyages to Antarctica and the Southern Ocean (Headland, 2009). To our knowledge, these current estimates are the first attempt to estimate total ship numbers and voyages for the latter part of the 20th Century or early 21st Century across all industries, though the tourism industry and the history of Antarctic voyages into the late 20th Century have been studied in detail (Bender et al., 2016; Headland, 1989, 1994, 2009; Lynch et al., 2010). Although the current estimates and how they were derived are presented here, further quantification of current ships and voyages, especially including the most popular destinations or pathways, is needed to assess likely sources for marine non‐native species.

**Table 2:** Estimates of the number of ships active around and voyages to the Antarctic region. For activity types where number of voyages is unknown (?) it is assumed that each vessel made at least one voyage to the Antarctic region. IUU = illegal, unreported and unregulated.

	Year	Number of vessels	Number of voyages
Research and resupply	2016–2017	52 (COMNAP, 2018b)	100+
Tourism (IAATO)	2017–2018	50 (IAATO, 2018b)	322 (IAATO, 2018a)
Tourism (non‐IAATO yachts)	2017–2018	19 authorised (United Kingdom, Argentina, Chile, & IAATO, 2018)	?
		9 unauthorised (United Kingdom, Argentina, Chile, & IAATO, 2018b)	?
Fishing (regulated)	2017–2018	47 (CCAMLR, 2018a)	?
Fishing (IUU)	Sighted since 2016	3 (CCAMLR, 2018b)	?
Total		183	~500+

**Figure 4:**
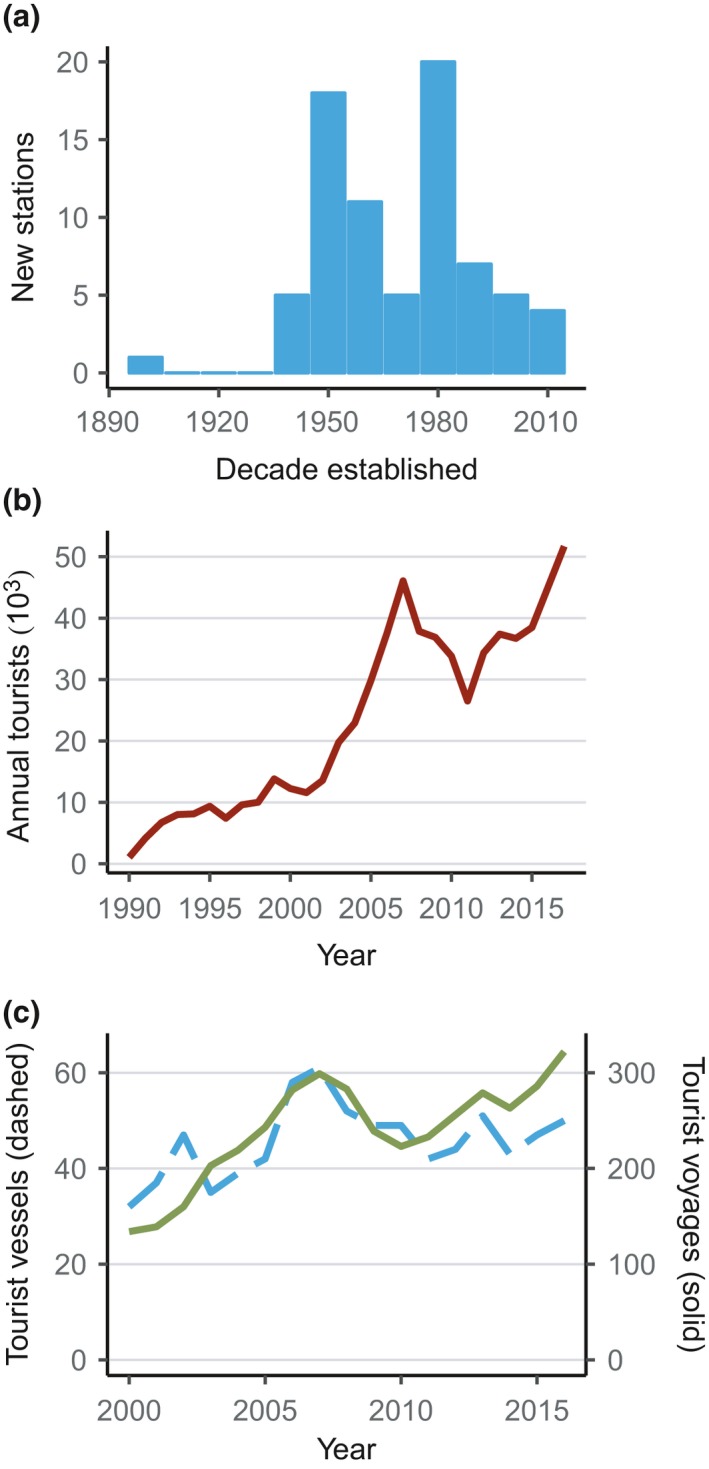
Changes in research and tourism activity in the Antarctic region over time. (a) Number of new Antarctic stations built per decade, data from COMNAP (2018a); (b) Number of tourists per year from the 1990–1991 to 2017–2018 austral summer seasons; (c) Number of tourism vessels per austral summer season between 2000–2001 and 2016–2017 active in Antarctica and the Southern Ocean (dashed) and number of tourist voyages per austral summer season to Antarctica and sub‐Antarctic islands (solid), data from IAATO (2018a)

Research and discovery have motivated Antarctic voyages since the 18th Century, but in the latter part of the 20th Century interest and activity from scientists increased rapidly. This lead up to the International Geophysical Year (IGY) in 1957/58 and the Antarctic Treaty (agreed 1959, entered into force 1961) resulted in the construction of many new research stations (Figure 4a; Headland, 2009). Another period of station‐building occurred in the 1980s (Figure 4a) as interest grew in Antarctic science and governance and many new Parties signed the Antarctic Treaty (Headland, 2009). A total of about 100 facilities, including 77 stations operated by 30 countries, are now in use in Antarctica (COMNAP, 2018a), albeit of varying scale and sophistication. Stations can accommodate anywhere from 10 to 1,300 people, many are seasonal or not necessarily used every year. Moreover, redevelopment of existing stations can require logistics equivalent to building a new station, including many ship visits. For example, Rothera Research Station wharf and building redevelopment required visits from a ship that would otherwise not have visited Antarctica. Nonetheless, most stations will require at least one visit and some multiple visits per season. Fifty‐two ships are currently used by national Antarctic programmes for research and resupply (COMNAP, 2018b), including some military vessels that are not subject to Antarctic Treaty regulations in the same way as national or tourist operators. Many ships will make multiple journeys to Antarctica or sub‐Antarctic islands within a season and given the number of research stations, there are probably at least 100 research and resupply voyages to Antarctica and sub‐Antarctic islands each year.

Similar to research activity, large‐scale Antarctic tourism increased in the late 20th Century. Tourists have visited sub‐Antarctic Islands since 1891 and the Antarctic continent since 1957 (Headland, 1994). Numbers initially grew slowly reaching approximately 1,000 tourists on 12 vessels in the 1990–1991 season (Headland, 1994; IAATO, 2018a). However, the industry has expanded rapidly since the 1990s (Figure 4b, 4c). In the 2017–2018 season, for the first time, over 50,000 tourists visited Antarctica on 50 vessels and 322 voyages operated by IAATO members (Figure 4b, 4c; Table 2). A decline in tourist numbers from 2008–2009 to 2012–2013 is attributed to the global financial crisis of 2007 (Bender et al., 2016), and numbers recovered to former levels by 2016–2017. The trend in the number of tourists is largely reflected in the number of tourist vessels operating in Antarctica as well as the number of voyages each season (Figure 4c). In addition, the International Maritime Organisation's ban on heavy fuel oil around Antarctica (IMO, 2010) will have affected which tourist ships could access Antarctica, excluding some larger vessels. Furthermore, at least 28 yachts visited the Antarctic region in 2017–2018 that were not operated by IAATO members (Table 2).

**Figure 5:**
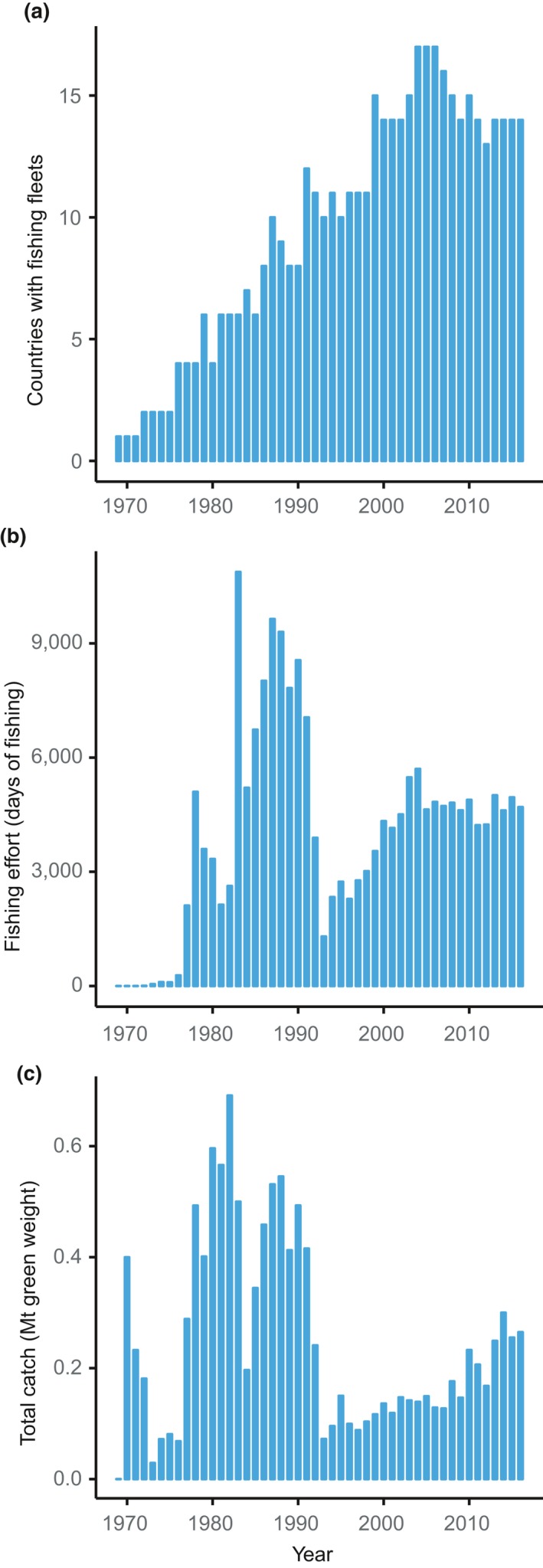
Fishing activity in the Southern Ocean since 1969. (a) Number of countries with fishing vessels in the Southern Ocean; (b) total number of fishing days for all vessels per year; (c) total catch (green weight – weight when caught) for all species per year. Data from CCAMLR (2018a)

Different again is the impact and activity of fishing vessels. Fishing pressure increased rapidly in the 1970s and 1980s and, after a decrease in the early 1990s, is going through a period of moderate expansion in number of fishing days and catch volume (Figure 5). Over time, target species, fishery locations and gear types have also changed, possibly altering the risk of NNMS introductions. Since 1999, 14–16 countries have had fishing fleets in CCAMLR areas (Figure 5a) and CCAMLR received approximately 90–150 requests for fishing vessels to operate in different areas in 2018 (Brooks et al., 2018), although a single vessel can fish multiple areas. For the 2017–2018 season, 47 fishing vessels were authorised to fish in CCAMLR areas (CCAMLR, 2018a) and three illegal, unregulated and unreported (IUU) vessels have been sighted since 2016 (16 since 2003) (CCAMLR, 2018b). During the 1990s and early 2000s IUU fishing took large unreported quantities of toothfish that may have exceeded the reported catch by four times (Agnew, 2000). Given the likely lack of adherence to international hull and ballast water biosecurity measures, IUU fishing vessels could pose a relatively high risk of NNMS introductions compared to non‐IUU fishing.

## 3 WHY ARE THERE CURRENTLY NO ESTABLISHED POPULATIONS OF NNMS IN THE ANTARCTIC REGION?

The very low number of NNMS recorded around Antarctica and sub‐Antarctic islands, i.e. overcoming stage 3 of the invasion pathway (Figure 1), might seem surprising given the history and traffic in the Southern Ocean. However, these low numbers could be caused, in part, by interactions between physical factors (ice cover, water temperature, seasonality, disturbance regimes) and biological factors (physiological limits, life history, Antarctic biodiversity) (Figure 1). Further to this, an incomplete understanding of Antarctic biodiversity and somewhat limited sampling effort, especially prior to the late 20th Century, may also be a factor. Nonetheless, for 15–30 million years, the Southern Ocean and coastal Antarctica have had limited dispersal from adjacent temperate ecosystems (Fraser et al., 2018; Peck, 2018; Zachos, Dickens, & Zeebe, 2008). The flow of the Antarctic Circumpolar Current (ACC) (Figure 3) forms a physical barrier that limits passive dispersal of new species to Antarctica (Clarke, Barnes, & Hodgson, 2005; Fraser et al., 2018). Shallow subsurface or floating materials may also be temporarily blocked by seasonal sea‐ice that surrounds the continent, increasing dispersal times. Moreover, individuals that manage to cross the Polar Front are confronted with ice in all its forms, freezing temperatures, physical disturbance from ice‐bergs, and strong seasonal variation in light availability and water chemistry. These extreme conditions often sit at or are beyond the physiological limits of potentially invading marine species (Aronson et al., 2007), and may limit the survival of non‐Antarctic species that reach Antarctica (Aronson, Frederich, Price, & Thatje, 2015; Byrne et al., 2016). The specialist adaptations to survive environmental extremes seen in native Antarctic species exemplify why the establishment of NNMS in the Antarctic region may be rare. Because the factors are so closely linked, the biological factors are discussed with each physical factor.

### 3.1 Ice and ice cover

Ice is perhaps the most pervasive factor constraining marine life around Antarctica and limits the availability of shallow‐subtidal habitat typically favoured by biofouling taxa (Peck, 2018). Ice, in addition to the implicit freezing temperatures, has direct, physical impacts on shallow water communities (Gutt, 2001). During winter, all the coastline and most of the ocean south of 60°S is affected by ice, much of which melts in the summer months to reveal stretches of coastline and ocean (Figure 2, February vs. September sea ice extent). Overall Antarctica has 2.3% of the world's coastline, but less than one seventh of this coastline is ice‐free in summer, and there is no ice‐free coastline in winter (Peck, 2018). The seasonal formation and melting of ice drives variation in salinity, disturbance regimes, light reaching the water column and seabed, and water currents. Moreover, the Antarctic ice‐cap depresses the continent, which combines with ice scour from previous geological periods, to create a deep continental shelf, with average depth between 400 and 500 m, compared to approximately 200 m depth elsewhere in the world (Peck, 2018). Since the potential colonizers in the coming decades are most likely to be biofouling species adapted to shallow water, the deep continental shelf may further limit the available habitat for potential colonizers, although this effect may be less important than the prevalence of ice cover or water temperature.

### 3.2 Freezing water temperatures

The Southern Ocean is characterized by cold, but stable, temperatures (Barnes, Fuentes, Clarke, Schloss, & Wallace, 2006) that pose severe physiological challenges to marine species. Minimum temperatures for shallow waters in winter range from freezing (approximately −1.85°C) around most of Antarctica to approximately −0.5°C at the most northerly islands, just south of the ACC (Barnes et al., 2006). Maximum temperatures in summer can be as warm as approximately 3°C along the northern parts of the Antarctic Peninsula but are typically closer to 0°C. The absence of decapod crustaceans such as brachyuran crabs, astacid lobsters and, to a lesser extent, anomuran king crabs from shallower Antarctic benthic shelf communities may be partly due to their limited capacity to regulate magnesium ions (Aronson et al., 2015; Barnhart, 2001; Griffiths, Whittle, Roberts, Belchier, & Linse, 2013; Wittmann, Held, Pörtner, & Sartoris, 2010) and reduced muscle power (a key characteristic of shell‐crushing predators) at cold temperatures (Aronson et al., 2007; Peck, 2018). Even the cold‐temperate waters (5–10°C) found around Punta Arenas in Chile, appear sufficiently cold to act as a barrier to non‐native ascidians in the Magellanic region (Turon et al., 2016), thereby making an invasion of Antarctica more unlikely.

Antarctic species possess adaptations to combat freezing body tissues and slower rates of biological processes associated with cold temperatures. Antarctic fish, particularly Notothenioids, produce antifreeze glycoproteins that inhibit their fluids from freezing (DeVries, 1971). Antarctic intertidal and subtidal invertebrates including limpets, marine mites, copepods and nemerteans typically do not produce antifreeze but are able to remain unfrozen at temperatures much lower than the freezing point of seawater (Waller, Worland, Convey, & Barnes, 2006). Biological processes, especially those involving protein synthesis and folding, are dramatically slowed because low temperature reduces protein stability and affects folding (Peck, 2016). To combat this, Antarctic marine species often produce more RNA and higher concentrations of heat‐shock proteins (Peck, 2018). Notothenioid fish possess an adaptation in their pectoral muscle to allow movement at speeds similar to tropical and temperate labriform swimmers. They have twice the mitochondria volume density (i.e. double the number of mitochondria per cm^3^ of muscle) in their red muscle tissue compared to species from warmer habitats (Johnston, Calvo, Guderley, Fernandez, & Palmer, 1998). The clam, *Laternula elliptica*, also compensates for reduced muscle capacity at low temperature, but it does this by having a foot muscle 2–3 times larger than temperate and tropical congeners (Morley, Lurman, Skepper, Pörtner, & Peck, 2009; Morley, Tan et al., 2009). Excepting these adaptations, no other examples of Antarctic species compensating for slowed biological processes have been reported to date. Antarctic marine animals are also characterized by long generation times and highly extended developmental times (Peck, 2018), and these are factors that would be expected to make them less competitive with NNMS invaders.

The extent to which NNMS would require specialized adaptations or characteristics to survive in the Antarctic region has received very little attention in past research and is unclear. Moreover, the heterogeneity of marine environments within the Antarctic region may allow species to establish in some areas while precluding them from others. Nonetheless, cold water temperatures in Antarctic environments seem to provide substantial physiological barriers to new introductions.

### 3.3 Disturbance regimes

Disturbance is a consistent feature of Antarctic shallow benthic environments due to the ice scour from icebergs and sea‐ice pressure ridges, which can affect shelf regions up to 500 m deep (Barnes & Conlan, 2007). Ice scour causes mortality in the underlying benthic community and creates patches of seabed at various stages of succession. Ice scour occurs most frequently in summer months but varies from year to year (Barnes, 2017; Barnes & Conlan, 2007; Barnes, Fenton, & Cordingley, 2014). It can affect 30%–95% of the seabed each year, depending on depth (25–5 m) (Barnes et al., 2014) and has been reported to remove over 99.5% of all macrofauna from the seabed (Peck, Brockington, Vanhove, & Beghyn, 1999). Immobile fauna, such as the bryozoan, *Fenestrulina rugula*, appear to have life histories that are adapted to the current frequency of ice scour (Barnes & Souster, 2011).

While ice scour may serve as a barrier to the establishment of some species, ice scour can also be a form of dispersal for Antarctic species because organisms on rocks and sediment that collect on the underside of a scouring ice‐berg and can be carried to new areas. Such a regular disturbance regime regularly clears new areas of seabed and hence could provide opportunities for NNMS that are often good colonizers of disturbed habitats.

### 3.4 Seasonality

High latitudes have strong seasonal cycles driven by extreme seasonal variation in photoperiod from winter to summer. Seasonality in Antarctic marine environments is reflected in relatively small temperature changes, formation and melting of sea‐ice, nearshore surface water salinity fluctuation from glacial run‐off and sea‐ice melt (Meredith et al., 2013), and short primary production peaks in spring and summer. The presence of sea‐ice in parts of the Antarctic region for much of the year also decreases light availability below the ice and decreases surface mixing, increasing stratification within the water column (Venables, Clarke, & Meredith, 2013). The common Antarctic sea urchin (*Sterechinus neumayeri*) and sea star (*Odontaster validus*) have higher oxygen consumption in summer when food is more abundant and when water temperatures are higher (Souster, Morley, & Peck, 2018). Feeding rates are high in summer months, while in winter, many species cease feeding (Barnes & Clarke, 1995). Overall, however, Antarctic species tend to have slow growth and very slow gamete and larval development (Peck, 2018).

## 4 CLIMATE CHANGE INCREASES THE RISK OF NNMS ESTABLISHING AROUND ANTARCTICA

Two key elements have limited the transport and establishment of NNMS in Antarctica (stage 3, Figure 1): relatively little human activity and inhospitable conditions. Yet, both these factors are changing. In particular, changes in physical factors (decreasing ice cover, increasing water temperature) will, if unchecked, in time create an environment more hospitable to non‐native species and less hospitable to native species, reducing the resistance of Antarctic ecosystems (Antarctic biodiversity) to establishment of NNMS (Figure 1). With climate change, seasonality will remain strong in Antarctic regions and may well limit the capacity of NNMS to establish. Meanwhile, ocean acidification will have uncertain effects. The future of each physico‐chemical environmental factor is discussed with reference to the biological factors of life history, physiological limits and Antarctic biodiversity.

### 4.1 Increasing water temperature

Increasing water temperature will likely improve the survivorship of NNMS and have a detrimental impact on some native communities, decreasing biotic resistance. Between 1955 and 1998, the summer sea‐surface waters off the north‐western Antarctic Peninsula warmed by ≳1.0°C (Meredith & King, 2005) and the northern fringes of the Southern Ocean are projected to warm by ≳1.0°C by 2100 (Gutt et al., 2015). In general, Antarctic invertebrates respond poorly to temperature increases, showing limited capacity for acclimation and phenotypic plasticity compared to temperate species (Ingels et al., 2012; Peck, Morley, Richard, & Clark, 2014). While an in situ change of 1°C was reported to increase recruitment and growth rates of some Antarctic encrusting fauna, rates decreased for other species, resulting in an altered community composition (Ashton, Morley, Barnes, Clark, & Peck, 2017). This study also showed that a warming of 2°C was close to, or above the long‐term survival temperature for some species.

Volcanically active and ice‐free marine habitats on the Antarctic Peninsula may already provide small areas that are suitable for non‐native species. At Deception Island in the South Shetland Islands, geothermal activity creates warm environments for terrestrial species along shorelines (Lewis Smith, 2005; Sturz, Gray, Dykes, King, & Radtke, 2003), and warm water temperature anomalies within the caldera (volcanic crater), especially in winter (Berrocoso et al., 2018). Compounding the effect of more favourable environmental conditions, Deception Island is amongst the most popular tourist landing sites, has two research stations and is considered both at highest risk and the most highly invaded location in the Antarctic Treaty area (south of 60°S) for terrestrial invasions (Chown et al., 2012). Deception Island may have already become a first entry point for intertidal or shallow‐water non‐native marine species (Table 1). The recent record of the brachyuran crab, *Halicarcinus planatus*, at Deception Island is its most southerly record (Aronson et al., 2015) and could be a sign of a southward range extension or inter‐regional transfer. *Halicarcinus planatus* is unusual because it reproduces in summer and winter and has juvenile stages that are tolerant of cooler temperatures (Diez & Lovrich, 2010).

If seabed temperatures warm sufficiently around Antarctica, crabs could establish with profound impacts; either through anthropogenic introduction of non‐native species (e.g. *H. araneus*, or *Carcinus maenas*), local deep‐water species extending their ranges to shallower waters, or range shifts by nearby sub‐Antarctic and temperate species (Griffiths et al., 2013). Shell‐crushing predators, such as *H. planatus*, are absent from shallow Antarctic shelf communities but have the potential to disrupt benthic ecosystems if they establish (Aronson et al., 2007; Griffiths et al., 2013). In the Arctic, predation by the introduced red king crab, *Paralithodes camtschaticus*, in the Barents Sea (bottom water 4–6°C) has decreased soft‐sediment benthic community biodiversity, altered sediment properties and changed benthic ecosystem functions (Oug et al., 2011, 2018).

### 4.2 Decreasing ice cover

Changes in terrestrial and marine ice cover are affecting Antarctic communities and will possibly increase the chance of NNMS establishing. Terrestrial Antarctica is predicted to become increasingly ice‐free throughout the century, particularly on the northern Antarctic Peninsula in Antarctic Conservation Biogeographic Regions (ACBRs) 1, 2 and 3 (Lee et al., 2017). These regions, due to warmer temperatures, increasing water availability, increasing ice‐free habitat and increased human visitation, have the highest risk of colonization by non‐native vascular plants (Chown et al., 2012; Hughes et al., 2015). In the marine realm, since 1957, sea‐ice extent and concentration in Antarctica increased in some areas while decreasing in others (Gutt et al., 2015; Steig et al., 2009), particularly the western Antarctic Peninsula (Steig et al., 2009). Moreover, ice shelves, especially on the Antarctic Peninsula are collapsing (Cook & Vaughan, 2010; Hogg & Gudmundsson, 2017) and exposing new areas of the seabed to higher light levels and changes in nutrient and propagule input. Native macroalgae have colonized newly uncovered shallow areas in the South Shetland Islands (Quartino et al., 2013). Changes in sea‐ice will not only affect benthic communities but will also impact pelagic krill‐based ecosystems (Clarke et al., 2007). Currently, ice‐scour of ships’ hulls may prevent transfer of non‐native species because the predominantly shallow, subtidal organisms are deposited far from shore and in deep water (Hughes & Ashton, 2017). However, with a decrease in ice cover, hull scraping may enhance propagule deposition as the organisms are removed from the hulls closer to shore and in shallower water.

Given some vessels active around Antarctica are also active in the Arctic, if a viable transport pathway between the Arctic and Antarctica were to exist, there is potential to transport ice‐tolerant taxa between the poles. For example, Arctic populations of periwinkles (e.g. *Littorina saxatilis*), barnacles (e.g. *Balanus balanoides*, *Chthamalus dalli*), blue mussels (*Mytilus edulis*), oysters (*Crassostrea virginica*) and clams (*Mya arenaria*) are fast growing and resistant to freezing and mechanical impacts of ice (Gutt, 2001). In addition, barnacles, blue mussels and oysters are known fouling species. Furthermore, balanomorph barnacles, such as *Balanus balanoides *and *Chthamalus dalli*, are absent from Antarctic regions (Peck, 2018). Should these species enter into invasion pathways to Antarctica and survive the journey, they may be capable of establishing in Antarctica and altering benthic ecosystems. However, the likelihood of Arctic species surviving a journey to Antarctica or successfully spreading from colonized areas of Southern Hemisphere regions is uncertain.

### 4.3 Strong seasonality

Antarctic environments will continue to experience extreme seasonal variation in light availability and a deep continental shelf compared to other parts of the world. However, light availability is affected by ice cover and UV‐B radiation is affected by ozone depletion, which combined are causing an increase in UV‐B radiation in near‐shore areas of the Antarctic Peninsula and the retreating ice edge (Gutt et al., 2015). Sea‐ice duration, via effects on light availability, determines the balance between benthic communities dominated by macroalgae or invertebrates (Clark et al., 2013). Duration of the sea‐ice season in waters around the Antarctic Peninsula decreased by 4 days per year from 1979/1980 to 2011/2012 (Hughes & Ashton, 2017). Further predicted increases in light availability will likely drive communities dominated by benthic fauna, e.g. sponges and corals, to those dominated by macroalgae (McClintock, Ducklow, & Fraser, 2008; Quartino et al., 2013) with significant consequences for regional biodiversity (Clark et al., 2013) and potentially creating habitat for non‐native species from macroalgae‐dominated communities elsewhere. Moreover, altered glacial activity is changing seasonal cycles of salinity and nutrient run‐off (Meredith & King, 2005; Meredith et al., 2013), both of which are biologically relevant to potential colonizers. Such turbulence in habitat and community composition may provide new opportunities for NNMS to establish in Antarctica.

### 4.4 Disturbance regimes

Decreasing sea‐ice duration and subsequent increasing disturbance from ice‐scour may both decrease community resistance to invasion and provide more available substrata for colonization. Decreases in sea‐ice duration combined with ice shelf collapse and glacial retreat are increasing the frequency of disturbance from ice‐scour in the western Antarctic Peninsula (Barnes, 2017). Moreover, ice‐scour in shallow depths (5–25 m) is negatively correlated with fast‐ice coverage (Barnes & Conlan, 2007) because fast‐ice locks icebergs in place. Increasing ice ‐scour is predicted to affect 61% of total Antarctic shelf areas, mainly in East Antarctica and the Antarctic Peninsula where sea‐ice duration is decreasing (Gutt et al., 2015). Biological consequences of more frequent disturbance from ice‐scour include increased mortality in native bryozoan colonies near Rothera Research Station (Barnes & Souster, 2011). Communities with greater biodiversity and high levels of competition are thought to be more resistant to non‐native species (Elton, 1958), yet increased ice‐scour in Antarctic shallow sites may decrease competition in benthic communities (Barnes et al., 2014), and thus increase vulnerability to invasions.

### 4.5 Ocean acidification

Future biofouling communities and potential colonizers will be determined, in part, by species’ tolerance of ocean acidification, adding a layer of complexity to the existing challenge of predicting potentially invasive species globally. Ocean acidification, and subsequent undersaturation of calcite, is a global and pervasive change in the marine environment (Kroeker et al., 2013) and it will affect all Antarctic marine ecosystems (Gutt et al., 2015). Increasing undersaturation of calcite worldwide will limit species’ capacities to produce calcified structures, increasing the relative costs of shell production compared to other processes (Watson, Morley, & Peck, 2017). This will have significant consequences, especially for biofouling communities: for example, a decreased proportion of encrusting (Spirorbid) worms and an increase in ascidians and sponges (Peck et al., 2015). The higher solubility of carbonate at low temperatures means the polar oceans may be particularly affected by undersaturation in the coming decades (Feely, Doney, & Cooley, 2009; Lebrato et al., 2016). This contributes to difficulties building external skeletons for taxa such as bivalves, gastropods and echinoderms, which are typically smaller and have thinner shells at high latitudes (McClintock et al., 2009; Watson et al., 2012) and for which shell‐building is costly (Watson et al., 2017). Moreover, lower pH may increase temperature sensitivity in crabs, such as *H. araneus *(Walther, Sartoris, Bock, & Pörtner, 2009), and impairs the neurotransmitter function in both vertebrates and invertebrates, with consequences for behaviour and predator–prey interactions (Chivers et al., 2014; Nilsson et al., 2012; Watson et al., 2013). Whether ocean acidification will increase or decrease the likelihood of NNMS establishing around Antarctica is uncertain.

## 5 INTERNATIONAL AGREEMENTS AND GOVERNANCE IN ANTARCTICA

**Figure 6:**
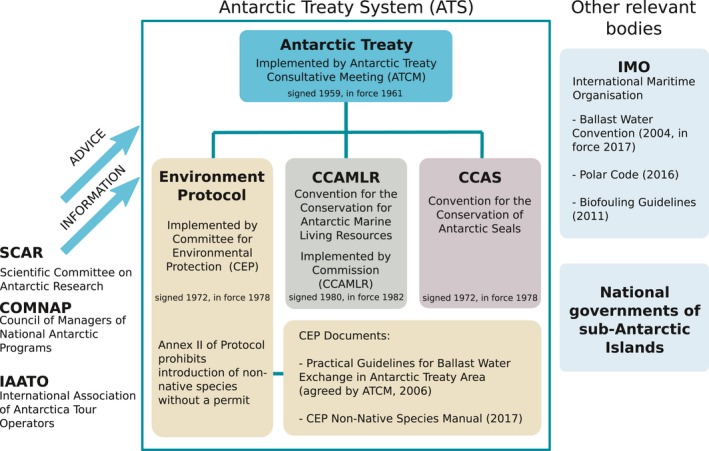
Antarctic Governance structure, including legislation, other international bodies and key documents relevant to the possible introduction and management of non‐native marine species in the Antarctic region

Unlike the effects of changing environmental conditions discussed above, the impact of some human activity in Antarctica can be directly managed through international agreements operating at most stages of the invasion process (Figure 1). Good, comprehensive governance has the potential to protect Antarctica from NNMS. Here, we outline the main regulatory frameworks relevant to NNMS in Antarctica (Figure 6).

The Antarctic Treaty System (ATS) comprises the Antarctic Treaty (entered into force 1961) and subsequent agreements. The ATS forms the primary legal and cooperative framework relating to human activity in and around Antarctica, specifically the area south of 60°S (Figure 6). The Antarctic Treaty area is governed through consensus by the 29 Consultative Parties to the Antarctic Treaty who have designated it as “… a natural reserve, devoted to peace and science” (Art. 2, ATCM, 1991). In particular, the Protocol on Environmental Protection to the Antarctic Treaty (Environmental Protocol or Madrid Protocol, entered into force in 1998; ATCM, 1991) ensures that activities in the Antarctic Treaty area are carried out with efforts to limit their environmental impacts, including banning deliberate introductions of non‐native species unless in accordance with a permit issued by an appropriate national authority (Art. 4, ATCM, 2009). The Committee for Environmental Protection (CEP) was established by the Environmental Protocol as an expert advisory body to provide advice and formulate recommendations to the ATCM in connection with the implementation of the Environmental Protocol. Additional bodies that can provide information to or advise the ACTM on science or human activity include: CCAMLR (manages fishing, part of the ATS), the Scientific Committee on Antarctic Research (SCAR; provides scientific advice to the ATCM), COMNAP (covers logistical activity and national operations, can advise the ATCM), and IAATO (covers most tourism, can provide information but not advice). Only the 53 nations that are signatories to the Antarctic Treaty have agreed to comply with ATS regulations (though not all nations have signed all components of the ATS). However, 174 member nations are required to comply with regulations from the International Maritime Organisation (IMO), including those relevant to Antarctic waters.

Since entering into force in September 2017, the IMO Ballast Water Management Convention (BWM) (IMO, 2004) has regulated the movement of NNMS via ballast water worldwide. It requires that ships treat and manage ballast water to reduce the chance of transferring potentially invasive species. The ATCM adopted the *Practical Guidelines for Ballast Water Exchange in Antarctic Waters* in 2006 (ATCM, 2006), which are best practice for Antarctic waters and request ballast water exchange at the Polar Front. These guidelines were later adopted by the IMO (IMO, 2007). In contrast to the BWM and Antarctic ballast water guidelines, no international requirements for hull‐fouling are in force, although the IMO Biofouling Guidelines (IMO, 2011) are a significant step towards consistent management of biofouling and minimizing introductions. In addition, the IMO Polar Code (IMO, 2016) includes that ships operating in polar regions should follow the Antarctic Ballast Water Guidelines (IMO, 2007) and the Biofouling Guidelines (IMO, 2011) as well as ensuring that ballast water management systems are suitable for polar conditions.

Biosecurity is the cornerstone of effective NNMS management. Since the early 2000s concern has grown over biosecurity measures in place for Antarctica, particularly for terrestrial environments (Chown et al., 2012; Hughes & Convey, 2010, 2012, 2014; Hughes & Frenot, 2015; Hughes et al., 2011; Lewis et al., 2003; McGeoch et al., 2015), and a decision‐making process for dealing with suspected introduced species has been formulated (Hughes & Convey, 2012). While response plans for eradication and management of non‐native terrestrial species are in development, the limited information about non‐native marine species has created difficulties for the development of marine‐focused policies (Hughes & Pertierra, 2016). Furthermore, it remains uncertain who, if anyone, could be held liable for introductions (Hughes & Convey, 2014). Non‐native species issues are considered one of the highest priority issues in the work of the CEP (CEP, 2017, 2018); however, progress on NNMS has been slowed due to a lack of research to inform decision‐making. Nevertheless, greater engagement with the issue of marine non‐native species is anticipated as more research comes to light.

The ATS and recent IMO agreements ensure that a number of invasion pathways common elsewhere in the world such as aquaculture, deliberate introductions, and pet or live seafood trade (Chan et al., 2019; Molnar et al., 2008) are not concerns for Antarctica. Moreover, the international agreements ensure guidelines are in place for the relevant vectors of ballast water and biofouling. However, it remains to be seen how closely biosecurity measures will be followed by operators or enforced by the national permitting authorities. Additionally, determining the native or non‐native status and likely vector of newly found species is a non‐trivial task, perhaps particularly so in Antarctica which is not as well studied as many parts of the world. Hughes and Convey (2012) discuss in detail methodologies for determining the native or non‐native status of newly observed terrestrial and freshwater species in Antarctica, much of which is relevant to marine species, too. A major concern is that there is insufficient evidence of natural dispersal patterns or anthropogenic propagule pressure to inform decisions, particularly for species that are transferred between areas within the Antarctic region or from South America.

## 6 THE FUTURE OF HUMAN ACTIVITY AND ANTHROPOGENIC MARINE INTRODUCTIONS

Human activity in Antarctica is increasing, especially since the latter part of 20th Century (Aronson et al., 2011; Bender et al., 2016), when tourism became increasingly popular, many research stations were built and fisheries expanded. The estimated 180 vessels and 500+ voyages annually in the Antarctic region represents a 5‐ to 10‐fold increase since the 1960s, based on estimates by Headland (1989, 2009). While many new ships will become operational in the near future to replace aging existing vessels (Witze, 2016), expansion of human activity in Antarctica shows little sign of slowing down, except where actively regulated.

The relatively recent expansion in human activity is particularly relevant given the lag time between a species’ introduction and subsequent detection (Crooks, 2005; Facon et al., 2006). Lag times and subsequent population explosions can be caused by adaptation to new environments, ecological changes in the new environment, or a combination thereof (Crooks, 2005; Facon et al., 2006; Lee, 2002; Prentis, Wilson, Dormontt, Richardson, & Lowe, 2008). For example, the Red King Crab population boom in the Barents Sea occurred 20–30 years after the initial introduction (Oug et al., 2011). Therefore, given the increase in human activity in Antarctica since the 1980s the possibility exists that NNMS are already in Antarctica, slowly adapting to conditions and poised to take advantage of ongoing environmental changes.

The impact of plastics on all aspects of the marine environment is growing and is the only non‐shipping vector discussed in this review. Antarctica, often thought of as pristine, is not isolated from plastic pollution with reported macroplastics and microplastics in the Southern Ocean beyond levels expected purely from Antarctic activity (Barnes, Walters, & Gonçalves, 2010; Waller et al., 2017). Marine plastics will augment the few natural rafting opportunities for biofouling organisms, although at high latitudes organisms appear unable to withstand the polar conditions (Barnes, 2002) and plastic debris is probably an unlikely vector for introductions to Antarctica (Lewis et al., 2005). Nonetheless, increases in plastic debris combined with increases in temperature and reductions in sea‐ice might make introductions via plastics more likely in the future.

## 7 RECOMMENDATIONS

This review has identified and discussed many factors that influence the risk of NNMS becoming established or invasive in Antarctica, yet detailed knowledge is lacking for many factors. To address some of the gaps identified and to facilitate improved management of the issues raised, we make the following recommendations for (1) researchers, (2) environmental managers, and (3) policy makers, with scope for input from stakeholders.

1. Researchers
  - Perform detailed analyses of records in databases such as OBIS and GBIF of species with distributions that include but are not limited to the Southern Ocean. Some NNMS may have already been transported to Antarctica, sub‐Antarctic islands and the Southern Ocean and may already have observations in these large, global databases which have not yet been reported as potential anthropogenic introductions in the Antarctic region.
  - Combine an assessment of current and projected ship activity (of all types) with habitat suitability for potential NNMS around Antarctica to identify areas that may become “invasion hotspots”
  - Improve models and predictions of future conditions along Antarctic coastlines to give better information on where to focus monitoring efforts for the early detection of newly establishing NNMS
  - Quantify propagule pressure from hull fouling and rank the relevant vectors and pathways to highlight those most at risk of introducing NNMS
  - Investigate the possible ecosystem impacts of NNMS around Antarctica, with consideration for how benthic NNMS may be relevant to predominantly pelagic Antarctic and Southern Ocean ecosystem models (Hill, Murphy, Reid, Trathan, & Constable, 2006; Murphy et al., 2012; Smith, Mincks, & Demaster, 2006)
  - Research issues related to transferring non‐native microorganisms or pathogens, particularly between fishing areas, because these are particularly poorly understood in the Antarctic region
2. Environmental managers
  - Identify and prioritize potential NNMS through formal horizon scanning and risk assessment (c.f. Roy et al., 2014)
  - Increase monitoring in high‐risk Antarctic marine environments, e.g. Deception Island and frequently visited areas of the Antarctic Peninsula, in light of new techniques such as eDNA
  - Increase monitoring in sub‐Antarctic marine environments, especially locations that are frequently visited en route to Antarctic locations and could act as stepping stones and bridgeheads for NNMS
  - Evaluate what, if any, marine‐specific biosecurity methods are used within the Antarctic region and their efficacy in the Antarctic context (e.g. in cold water)
3. Policy makers
  - Adapt to marine ecosystems the suggested methodology and conceptual frameworks already developed for managing non‐native species in Antarctic terrestrial and freshwater ecosystems (Galera et al., 2018; Hughes & Convey, 2012)
  - Develop rapid response plans for high risk NNMS
  - Undertake regular assessment of progress on the issue of NNMS within relevant organizations, e.g. ATCM, CEP, COMNAP, CCAMLR, and IAATO
  - Improve interaction between ATCM, CEP, COMNAP, CCAMLR, IAATO and Parties to make progress on the issues above, including sharing relevant data to allow other recommendations to be carried out.

## 8 CONCLUSIONS

Antarctica is undoubtedly experiencing significant ecological and environmental change, which may facilitate new species establishing. In particular, the effects of increasing water temperature, decreasing ice cover and increasing ship activity appear to be very important factors increasing the likelihood of NNMS establishing within the Antarctic region in the coming decades. These factors, in addition to their primary effects, interact with many of the other factors discussed in this review (Figure 1). Marine invasive species worldwide have social, economic and ecological consequences (Bax et al., 2003) and interact with other anthropogenic impacts such as climate warming, ocean acidification and pollution to intensify pressure on ecosystems (Aronson et al., 2011). However, the risk of introducing non‐native marine species to Antarctica or their potential impacts on existing ecosystems remains largely unquantified. Without research addressing the factors influencing the likelihood of marine biological invasions, it will be difficult to make evidence‐based management decisions. The Climate Change Response Work Programme of the CEP (CEP, 2015), identifies the potential introduction of NNMS to Antarctica as a priority for future research. This review contextualises current knowledge on the topic and provides recommendations for future research. Given climate change and increasing human pressure, Antarctica will not remain the untouched, pristine habitat of its reputation and any holistic view of Antarctica's future should consider the prospect of marine non‐native species becoming established.
