# Supplementary material for: Antarctica: The final frontier for marine biological invasions
Source: Glob Chang Biol. 2019 Apr 23;25(7):2221–41. doi: 10.1111/gcb.14600 (PMC6849521; doi:10.1111/gcb.14600)
Supplement: Supplementary file 1 [file GCB-25-2221-s001.docx]

**Supporting Information – Table S1**

Non-native marine species in pathways to Antarctica. Species listed have been found fouling ships travelling to Antarctica. Taxa that were reported but not identified to genus or species have not been included in this list. Species are listed using the most recent accepted name in the World Register of Marine Species (Horton et al., 2018), which may differ from the name in the original publication. Ships that have been surveyed multiple times, with multiple occurrences of the same species are only counted once, but all sources referenced.

* indicates that species may be higher risk due to known range in the Arctic, sub-Antarctic and/or tolerance of cold conditions. Underlined species are invasive in part of their range based on listing in the Global Invasive Species Database (Invasive Species Specialist Group ISSG, 2015). Global Range is for species only and based on distributions in the Ocean Biogeographic Information System (OBIS, 2018) and the Global Biodiversity Information Facility (GBIF: The Global Biodiversity Information Facility, 2018).

| **Taxa** | | **Number of ships reporting species (Source)** | **Country of gateway port** | **Evidence of invasiveness** | **Global Range** |
| --- | --- | --- | --- | --- | --- |
| **PLANTAE (kingdom)** | | | | | |
| *** | *Ulva intestinalis* Linnaeus, 1753 (gutweed, grass kelp) | 6 (Lee & Chown, 2009; Lewis, Riddle, & Hewitt, 2004; Lewis, Riddle, & Smith, 2005) | South Africa, Australia |  | Cosmopolitan except high latitudes. Cryptogenic in Antarctica |
| *** | *Ulva rigida* C.Agardh, 1823 | 3 (Lewis et al., 2004, 2005) | Australia |  | Cosmopolitan except high latitudes, although records from Iceland, Alaska and Macquarie Island (sub-Antarctic) |
| *** | *Ulva compressa* Linnaeus, 1753 | 2 (Lewis et al., 2004, 2005) | Australia |  | Cosmopolitan, except Antarctic and sub-Antarctic. Records from Canadian Arctic, Alaska, Iceland, Norway, Barents Sea and New Zealand sub-Antarctic islands. |
|  | *Ulva sp.* Linnaeus, 1753 | 4 (Lee & Chown, 2009; Lewis, Hewitt, Riddle, & McMinn, 2003) | South Africa, Australia |  |  |
|  | *Grateloupia filicina* (J.V.Lamouroux) C.Agardh, 1822 | 1 (Lee & Chown, 2009) | South Africa |  | Cosmopolitan except high latitudes. |
| *** | *Ectocarpus siliculosus* (Dillwyn) Lyngbye, 1819 | 1 (Lee & Chown, 2009) | South Africa |  | Mid to high latitudes, including NZ sub-Antarctic islands, Iceland, Barents Sea but excluding North American Arctic and Antarctica. |
|  | *Ceramium sp.* Roth, 1797 | 1 (Lee & Chown, 2009) | South Africa |  |  |
|  | *Cladophora sp.* Kützing, 1843 | 1 (Lewis et al., 2003) | Australia |  |  |
|  | *Cladophoropsis sp.* Børgesen, 1905 | 1 (Lewis et al., 2004) | Australia |  |  |
|  | *Bryopsis sp.* J.V.Lamouroux, 1809 | 1 (Lewis et al., 2004) | Australia |  |  |
|  | *Ruppia megacarpa*(Mason, 1967) | 1 (Lewis et al., 2004) | Australia |  | Southern Australia and New Zealand |
| **ANAMALIA (kingdom)** | | | | | |
| **Annelida (phylum)** | | | | | |
| **Polychaeta (class)** | | | | | |
|  | *Hydroides ezoensis* Okuda, 1934 | 1 (Lewis et al., 2004, 2005) | Australia | Introduced in North Sea, Australia, Britain, France (Atlantic) (Çinar, 2013) | NW Pacific, SE Australia, Europe. Native to N Pacific |
|  | *Hydroides elegans* (Haswell, 1883) | 1 (Lewis et al., 2005) | Australia | Widely introduced. (Çinar, 2013) | Widespread in low-mid latitudes. Native to Indo-Pacific. Introduced elsewhere. |
|  | *Sabella spallanzanii* (Gmelin, 1791)  European fan worm | 1 (Lewis et al., 2005) | Australia | Invasive in Australia (Çinar, 2013; Invasive Species Specialist Group ISSG, 2015) | Europe primarily. Southern Australia, New Zealand, few records in NW Pacific, South Africa, southern Argentina. |
|  | *Parasabella leucaspis* (Kinberg, 1867) | 1 (Lewis et al., 2005) | Australia | Invasive in Australia  (Çinar, 2013) | Australia? Few records scattered around. Possibly introduced from Peru |
|  | *Spirobranchus taeniatus* (Lamarck, 1818) | 2 (Lewis et al., 2003, 2004) | Australia |  | Australia and New Zealand. |
|  | *Polydora sp.* Bosc, 1802 | 1 (Lewis et al., 2003) | Australia | Many species considered invasive (Çinar, 2013) | Widespread genus with many species |
|  | *Harmothoe sp.* Kinberg, 1856 | 1 (Lewis et al., 2004) | Australia | *Harmothoe imbricate* (Linnaeus, 1767) introduced to USA (Atlantic) (Çinar, 2013) |  |
| **Arthropoda (phylum)** | | | | | |
| **Hexanauplia (class)** | | | | | |
| **Sessilia (order)** | | | | | |
|  | *Amphibalanus Amphitrite* (Darwin, 1854) | 3 (Lewis et al., 2004, 2005) | Australia |  | Cosmopolitan low-mid latitudes. |
|  | *Amphibalanus variegatus*(Darwin, 1854) | 1 (Lewis et al., 2004) | Australia |  | SE Asia and Oceania. |
|  | *Austrominius modestus* (Darwin, 1854) | 4 (Lewis et al., 2003, 2004, 2005) | Australia |  | Europe. SE Australia and New Zealand. |
| **Lepadiformes (order)** | | | | | |
|  | *Lepas sp.* Linnaeus, 1758 | 2 (Lee & Chown, 2009; Lewis et al., 2003) | South Africa, Australia |  |  |
| *** | *Conchoderma auritum* (Linnaeus, 1767) | 2 (Hughes & Ashton, 2017; Lewis et al., 2004) | (Europe via) South America |  | Cosmopolitan including Barents Sea, Alaska, South Georgia and South Sandwich Islands. |
| Malacostraca (Class) | | | | | |
| **Decapoda (order)** | | | | | |
|  | *Halicarcinus quoyi* (H. Milne Edwards, 1853) | 2 (Lewis et al., 2003, 2004, 2005) | Australia |  | SE Australia, New Zealand, including NZ sub-Antarctic Islands |
|  | *Macrobrachium sp.* Spence Bate, 1868 | 1 (Lewis et al., 2004) | Australia |  |  |
|  | *Palaemon serenus*(Heller, 1862) | 1 (Lewis et al., 2003) | Australia |  | Eastern and southern Australia. |
| **Amphipoda (Order)** | | | | | |
|  | *Monocorophium acherusicum* (Costa, 1853) | 2 (Lewis et al., 2004, 2005) | Australia |  | Widespread mid-high latitudes. |
|  | *﻿Monocorophium insidiosum* (Crawford, 1937) | 2 (Lewis et al., 2005) | Australia |  | Widespread in mid latitudes. |
|  | *Caprella sp.* Lamarck, 1801 | 1 (Lewis et al., 2003) | Australia |  |  |
| *** | *Jassa falcata* (Montagu, 1808) | 1 (Lewis et al., 2004) | Australia |  | Widespread, including Arctic, sub-Antarctic islands and Arctic continent. |
|  | *Jassa herdmani*(Walker, 1893) | 1 (Lewis et al., 2004) | Australia |  | British Isles, Europe. |
|  | *Jassa sp.* Leach, 1814 | 1 (Lewis et al., 2004) | Australia |  |  |
| Bryozoa | | | | | |
| *** | *Bugula neritina* Linnaeus, 1758 (brown bryozoan, common bugula) | 3 (Lewis et al., 2004, 2005) | Australia | Invasive in Argentina, Atlantic - Western Central, Australia, Belgium, Bermuda, Brazil, Chile, China, Ecuador, Egypt, France, Germany, India, Israel, Italy, Japan, North Korea, South Korea, Libyan Arab Jamahiriya, Mediterranean & Black Sea, Mexico, Netherlands, New Zealand, Panama, Philippines, Puerto Rico, Spain, Turkey, United Kingdom, United States (Invasive Species Specialist Group ISSG, 2015) | Cosmopolitan except high latitudes (native range unknown) |
|  | *Bugulina flabellate* (Thompson in Gray, 1848) | 3 (Lewis et al., 2004, 2005) | Australia |  | Primarily Europe. Australia, southern Africa, NW Atlantic. |
|  | *Bugulina stolonifera* (Ryland, 1960) | 1 (Lewis et al., 2005) | Australia |  | Widespread in low-mid latitudes. |
| *** | *Membranipora membranacea* (Linnaeus, 1767) | 1 (Lewis et al., 2004, 2005) | Australia |  | Europe, including Norwegian Arctic and Barents Sea. North America, Oceania, few records elsewhere in low-mid latitudes. |
|  | *Tricellaria occidentalis* (Trask, 1857) | 3 (Lewis et al., 2004, 2005) | Australia |  | Western Pacific, NE Pacific. |
|  | *Watersipora subtorquata*(d'Orbigny, 1852) | 5 (Lewis et al., 2003, 2004, 2005) | Australia | Considered alien in much of its range, but invasiveness uncertain or unspecified (Invasive Species Specialist Group ISSG, 2015) | Oceania, western Pacific, NE Pacific, |
| *** | *Schizoporella unicornis*(Johnston in Wood, 1844) | 1 (Lewis et al., 2005) | Australia | Invasive in Australia, Unspecified or uncertain invasiveness in Europe and Americas (Invasive Species Specialist Group ISSG, 2015) | Northern Europe, Svalbard, Barents Sea, North America, Oceania, Mediterranean, Red Sea. Native range in Japan/NW Pacific. |
|  | *Celleporina sp* Gray, 1848 | 1 (Lewis et al., 2004) | Australia |  |  |
|  | *Lagenorhynchus sp.* Gray, 1846 | 1 (Lewis et al., 2004) | Australia |  |  |
| Chordata | | | | | |
| *** | *Ascidiella aspersa* (Müller, 1776)  European sea squirt | 1 (Lewis et al., 2005) | Australia | Invasive in Australia, New Zealand, India, United States (Invasive Species Specialist Group ISSG, 2015) | Europe, primarily. Barents Sea. Widespread in mid-latitudes. Native range in NE Atlantic - Europe, Mediterranean. |
|  | *Styela plicata* (Lesueur, 1823) | 1 (Lewis et al., 2005) | Australia | Invasive in Australia. Uncertain or unspecified invasiveness elsewhere outside native range (Invasive Species Specialist Group ISSG, 2015) | Widespread in mid-latitudes. Native to Western-Central Atlantic |
|  | *Styela canopus* | 1 (Lewis et al., 2003) | Australia |  | Widespread in mid-latitudes. |
| *** | *Ciona intestinalis* (Linnaeus, 1767) (vase tunicate) | 4 (Lee & Chown, 2009; Lewis et al., 2003, 2005) | South Africa, Australia | Invasive in Canada, Chile, New Zealand, South Africa, United States. Uncertain or unspecified elsewhere outside native range (Invasive Species Specialist Group ISSG, 2015) | Widespread (North Atlantic, Mediterranean) |
|  | *Botrylloides leachii* (Savigny, 1816) | 1 (Lewis et al., 2003, 2005) | Australia |  | Europe and Mediterranean, Oceania. |
|  | *﻿Botryllus schlosseri* (Pallas, 1766) | 1 (Lewis et al., 2005) | Australia |  | Europe, North America, western Pacific. |
| Cnidaria | | | | | |
|  | *Bougainvillia muscus*(Allman, 1863) | 1 (Lewis et al., 2004, 2005) | Australia |  | Low to mid-latitudes. Primarily northern Europe. |
| *** | *Clytia hemisphaerica* (Linnaeus, 1767) | 2 (Lewis et al., 2004, 2005) | Australia |  | Cosmopolitan, including Svalbard. |
| *** | *Obelia dichotoma* (Linnaeus, 1758) | 3 (Lee & Chown, 2009; Lewis et al., 2004, 2005) | South Africa, Australia |  | Cosmopolitan, including Alaska, Canadian Arctic, Barents Sea, Svalbard. Excluding Antarctica and sub-Antarctic. |
|  | *Obelia sp.* Péron & Lesueur, 1810 | 1 (Lewis et al., 2004) | Australia |  |  |
| *** | *Ectopleura crocea*  (Agassiz, 1862) | 3 (Lewis et al., 2005) | Australia |  | Widespread in mid-latitudes. Records in Antarctica. |
|  | *Ralpharia magnifica* (Watson, 1980) | 2 (Lewis et al., 2003) | Australia |  | SE Australia. |
| Mollusca | | | | | |
| *** | *Mytilus galloprovincialis* Lamarck, 1819 | 6 (Lee & Chown, 2007; Lewis et al., 2003, 2005) | South Africa, Australia | Invasive in North America and southern Africa (Invasive Species Specialist Group ISSG, 2015) | Widespread in mid-latitudes. Few records in low latitudes. Present in sub-Antarctic islands |
|  | *Magallana gigas* (Thunberg, 1793) | 1 (Lewis et al., 2005) | Australia | Invasive in Atlantic - NE, Australia, Canada, Chile, Netherlands, New Zealand, South America, Unites States (Invasive Species Specialist Group ISSG, 2015) | Native range in NW Pacific. Widespread in mid-latitudes. |

**References:**

Çinar, M. E. (2013). Alien polychaete species worldwide: Current status and their impacts. *Journal of the Marine Biological Association of the United Kingdom*, *93*(5), 1257–1278. https://doi.org/10.1017/S0025315412001646

GBIF: The Global Biodiversity Information Facility. (2018). What is GBIF? Retrieved November 9, 2018, from https://www.gbif.org/what-is-gbif

Horton, T., Kroh, A., Ahyong, S., Bailly, N., Boyko, C. B., Brandão, S. N., … Zhao, Z. (2018). World Register of Marine Species (WoRMS). WoRMS Editorial Board. Retrieved from http://www.marinespecies.org

Hughes, K. A., & Ashton, G. V. (2017). Breaking the ice : the introduction of biofouling organisms to Antarctica on vessel hulls. *Aquatic Conservation: Marine and Freshwater Ecosystems*, *27*, 158–164. https://doi.org/10.1002/aqc.2625

Invasive Species Specialist Group ISSG. (2015). The Global Invasive Species Database. Retrieved November 9, 2018, from http://www.iucngisd.org/gisd/

Lee, J. E., & Chown, S. L. (2007). Mytilus on the move: Transport of an invasive bivalve to the Antarctic. *Marine Ecology Progress Series*, *339*, 307–310. https://doi.org/10.3354/meps339307

Lee, J. E., & Chown, S. L. (2009). Temporal development of hull-fouling assemblages associated with an Antarctic supply vessel. *Marine Ecology Progress Series*, *386*, 97–105. https://doi.org/10.3354/meps08074

Lewis, P. N., Hewitt, C. L., Riddle, M., & McMinn, A. (2003). Marine introductions in the Southern Ocean: An unrecognised hazard to biodiversity. *Marine Pollution Bulletin*, *46*(2), 213–223. https://doi.org/10.1016/S0025-326X(02)00364-8

Lewis, P. N., Riddle, M. J., & Hewitt, C. L. (2004). Management of exogenous threats to Antarctica and the sub-Antarctic Islands: Balancing risks from TBT and non-indigenous marine organisms. *Marine Pollution Bulletin*, *49*(11–12), 999–1005. https://doi.org/10.1016/j.marpolbul.2004.07.001

Lewis, P. N., Riddle, M. J., & Smith, S. D. A. (2005). Assisted passage or passive drift: a comparison of alternative transport mechanisms for non-indigenous coastal species into the Southern Ocean. *Antarctic Science*, *17*(2), 183–191. https://doi.org/10.1017/S0954102005002580

OBIS. (2018). Ocean Biogeographic Information System. Retrieved November 9, 2018, from www.iobis.og
